# IL1R2, CCR2, and CXCR4 May Form Heteroreceptor Complexes with NMDAR and D2R: Relevance for Schizophrenia

**DOI:** 10.3389/fpsyt.2017.00024

**Published:** 2017-02-15

**Authors:** Dasiel O. Borroto-Escuela, Alexander O. Tarakanov, Karl Bechter, Kjell Fuxe

**Affiliations:** ^1^Department of Neuroscience, Karolinska Institutet, Stockholm, Sweden; ^2^Department of Biomolecular Science, Section of Physiology, Campus Scientifico Enrico Mattei, University of Urbino, Urbino, Italy; ^3^Observatorio Cubano de Neurociencias, Grupo Bohío-Estudio, Yaguajay, Cuba; ^4^Russian Academy of Sciences, St. Petersburg Institute for Informatics and Automation, Saint Petersburg, Russia; ^5^Clinic for Psychiatry and Psychotherapy II, Ulm University, Bezirkskrankenhaus Günzburg, Günzburg, Germany

**Keywords:** receptor–receptor interactions, schizophrenia, neuroinflamation, NMDAR, chemokine receptors, cytokine receptors, heteroreceptor complexes, volume transmission

## Abstract

The mild neuroinflammation hypothesis of schizophrenia was introduced by Bechter in 2001. It has been hypothesized that a hypofunction of glutamatergic signaling *via N*-methyl-D-aspartate receptors (NMDARs) and hyperactivation of dopamine D2 receptors play a role in schizophrenia. The triplet puzzle theory states that sets of triplet amino acid homologies guide two different receptors toward each other and contributes to the formation of a receptor heteromer. It is, therefore, proposed that putative NMDAR-C-C chemokine receptor type 2 (CCR2), NMDAR-C-X-C chemokine receptor type 4 (CXCR4), and NMDAR- interleukin 1 receptor type II (IL1R2) heteromers can be formed in the neuronal networks in mild neuroinflammation due to demonstration of Gly-Leu-Leu (GLL), Val-Ser-Thr (VST), and/or Ser-Val-Ser (SVS) amino acid homologies between these receptor protomers. This molecular process may underlie the ability to produce symptoms of schizophrenia in mild neuroinflammation. In this state, volume transmission (VT) is increased involving increased extracellular vesicle-mediated VT from microglia and astroglia. These vesicles may contain CCR2, CXCR4, and/or IL1R2 as well as their ligands and upon internalization by endocytic pathways into neurons can form heteroreceptor complexes with NMDAR in the plasma membrane with pathological allosteric receptor–receptor interactions involving increased internalization and reduced NMDAR signaling. The triplet puzzle theory also suggests the formation of putative D2R-CCR2, D2R-CXCR4, and D2R-IL1R2 heteromers in mild neuroinflammation in view of their demonstrated sets of Leu-Tyr-Ser (LYS), Leu-Pro-Phe (LPF), and/or Ser-Leu-Ala (SLA) triplet homologies. These D2R heteroreceptor complexes may also contribute to schizophrenia-like symptoms in mild neuroinflammation by enhancing D2R protomer function.

## Introduction

The mild neuroinflammation hypothesis of schizophrenia was introduced by Bechter (1–3). Recent work supports a role of inflammation in schizophrenia (4) and a relevant cellular basis appears to be microglia, which upon activation release proinflammatory cytokines (4, 5). It is still unclear, however, if the classical antibiotic drug minocycline can be used as an antipsychotic drug in spite of its ability to block microglia activation. It is known that microglia plays an important role in brain development and possesses protective and destructive functions in neuroinflammation (6–8).

CSF studies may be especially informative on brain events, at least in the clinical situation, and can be performed repeatedly even during acute psychotic episodes. Such studies demonstrated, both in affective and schizophrenic spectrum disorders, the prevalence of activated CSF cells similar to the situation in neurological neuroinflammatory disorders (9, 10). In addition, at least three immunological subgroups were found in affective and schizophrenic spectrum disorders as defined by established CSF examination (11) and a subgroup with increased CSF neopterin (12). In another study, all patients investigated demonstrated an increase of IL8 (13). Taken together, between 70 and 100% of severely diseased patients with the affective and schizophrenic spectrum disorder presented certain CSF abnormalities (14, 15). These findings supported the mild encephalitis (ME) hypothesis of these disorders (1, 2, 16). Further support came from the neurological field with the first description of NMDAR autoimmune encephalitis (17, 18). The more general relevance of the ME hypothesis is suggested by epidemiological findings demonstrating that infections and autoimmune disorders are important risk factors in schizophrenia bipolar and depressive disorders (19–21). Furthermore, recent CSF studies in larger groups of similar psychiatric patient groups also supported this view (22–24).

There is an agreement that the *N*-methyl-d-aspartate receptor (NMDAR) hypofunction plays an important role in the schizophrenia disease development (25). Previously it was found that chronic brain inflammation can produce a decline in both hippocampal GluN1 NMDARs and GluN2A and GluN2B subunits of NMDARs which likely is mainly caused by reductions in their transcriptional mechanisms (26, 27). They may be linked to cognitive deficits is schizophrenia.

It is, therefore, of high interest that schizophrenia-like symptoms can often be found in patients with NMDAR antibody induced encephalitis (17, 19, 28–30). The NMDAR autoantibodies have been shown to lead to specific, titer-dependent, reversible loss of NMDARs, the dysbalance within the network function being able to explain a spectrum of symptoms (31). It seems possible that the mechanism can involve disturbances in NMDAR function through interactions with the NMDAR antibody leading *inter alia* to NMDA receptor internalization and breakdown (31). On the other hand, a broad repertoire of antibody-secreting cells is enriched in the CNS during encephalitis producing different types of autoantibodies in parallel in the CSF (32). In addition, for neuronal damage in autoimmune encephalitis cytotoxic, T cells may be responsible not the autoantibodies. Triggers of autoimmune encephalitis may be cancers or virus infections or remain unknown (33). So far, the situation is rather similar to that predicted with the ME hypothesis. The latter is further supported by CSF findings (14, 15) and not least by rare cases of acute psychosis with brain biopsy showing definite but mild neuroinflammation in the cerebral cortex (34–36). Recent evidence for a more general relevance of the ME hypothesis comes also from a postmortem study showing an increased number of immune cells in the brain seemingly linked to a minor blood brain barrier breakdown (37).

Apparently, in classical and ME, one can plausibly expect some general pathological mechanisms but potentially also specific pathological mechanisms to be involved in parallel. There exists no clearcut evidence that specific autoimmunity explains the whole disorder in autoimmune encephalitis (38) nor that it represents an exclusive single pathological mechanism. In multiple sclerosis (MS), there is an early involvement of the cerebral cortex found in both experimental allergic encephalomyelitis (39) and in human MS (40). The interesting findings by Najjar et al. (34–36) in cortical biopsies may similarly represent not only proof for mild local neuroinflammation but may in addition indicate a more distributed mild neuroinflammatory process, the latter indicated by the findings of Bogerts et al. (37).

The current perspective article will discuss the different molecular mechanisms that may underlie the ability of neuroinflammation to produce positive, negative, and/or cognitive symptoms of schizophrenia. It likely involves the release of chemokines and cytokines from activated microglia, astroglia, and monocytes (41, 42), which *via* volume transmission (VT) can target their receptors on glia and neurons (43, 44). There may also exist an increased extracellular vesicle-mediated VT (44) from glia and megacaryocytes. Glial and immune cells may contain receptor proteins and different forms of mRNAs for chemokine and cytokine receptors in mild neuroinflammation. Extracellular vesicles containing mRNA and proteins for these receptors can *via* VT communication have a relevant role for producing schizophrenia-like symptoms by being internalized *via* e.g., cell adhesion receptors into the neuronal component of glia–neuron networks. Extracellular vesicles containing e.g., the cytokine and chemokine receptors may be taken up by an uptake mechanism that depends on proteins located both on the neuronal target cell at extrasynaptic and/or synaptic sites and on the extracellular vesicle (45). The extracellular vesicles are then internalized by a number of endocytic pathways. In this process, the internalized receptors can reach e.g., early endosomes and be rapidly returned to the plasma membrane (46) where they are proposed to interact with extrasynaptic and synaptic NMDARs and D2Rs, indicated to be involved in schizophrenia. This may lead to pathological receptor–receptor interactions in neurons in brain areas with mild neuroinflammation (43, 44).

The allosteric receptor–receptor interactions in D2R heterocomplexes are already indicated to play a role in schizophrenia, especially the antagonistic A2AR–D2R interactions in A2AR–D2R heterocomplexes (47–49). Using the triplet puzzle theory (50), four sets of triplet amino acid homologies were found between the A2AR and D2R protomers which may contribute to the formation of the A2AR–D2R heterocomplexes and to the development of the antagonistic A2A–D2 receptor–receptor interactions (47–49).

## Possible Molecular Mechanisms Based on the Triplet Puzzle Theory Contributing to Schizophrenia-Like Symptoms in Mild Neuroinflammation

### Triplet Puzzle Theory Supports the Formation of Glutamate NMDAR–CytokineR/ChemokineR Heteroreceptor Complexes through Gly-Leu-Leu (GLL), Val-Ser-Thr (VST), and Ser-Val-Ser (SVS) Homologies

In 2010, based on a bioinformatic approach, it was possible to indicate that receptor that form heterodimers show triplet amino acid homologies (50). This was not observed in pairs of receptors that do not form heterodimers. It was, therefore, proposed that these triplet homologies participate in the receptor interface and gives a code that facilitates the formation of the heterodimer. It was named the triplet puzzle theory (50, 51). The code formed from the triplet amino acid homologies may assist in guiding the receptors toward each other.

Such protriplet homologies appear to be phylogenetically old mechanisms for protein recognition and are already found in integrins (an alpha–beta heterodimer) of marine sponges (52) and remain in human D2 receptor heteromers (53).

It is of particular interest that the NMDAR shows one protriplet amino acid homology with CCR2 (GLL), C-X-C chemokine receptor type 4 (CXCR4) (VST), and interleukin 1 receptor type II (IL1R2) (SVS) (Table 1) as previously observed (43). The GLL protriplet of CCR2 is located in the C-tail and may interact with the GLL of the intracellular part of NR2A (Table 1). The VST protriplet of CXCR4 is also found in the C-tail and may interact with the VST protriplet of the intracellular part of NR2A (Table 1). The VST protriplet of CXCR4 may also interact with the VST in the NR1-1,4,5 subunits present in the C-tail (Table 1). The SVS protriplet is located in the N-terminal of IL1R2 and may interact with the SVS protriplet in the extracellular part of NR2A,B,D (Table 1).

**Table 1:** **Example of schizo triplets in the interface of human receptor heteromers**.

Receptor heteromer	Reference	Ser-Val-Ser (SVS)	Gly-Leu-Leu (GLL)	Val-Ser-Thr (VST)	Leu-Tyr-Ser (LYS)	Leu-Pro-Phe (LPF)	Ser-Leu-Ala (SLA)
GABAB1–GABAB2	(54–56)	−	#	−	−	−	−
GABAB1–CXCR4	(57)	#	−	#	−	−	−
NMDA–CCR2	Possible heteromer	−	#	−	+	−	−
NMDA-interleukin 1 receptor type II (IL1R2)	Possible heteromer	#	−	−	−	−	−
NMDA–CXCR4	Possible heteromer	+	−	#	+	−	−
MOP–DOP	(58)	−	−	−	#	#	−
CCR2–CXCR4	(59)	−	−	−	#	−	−
DOP–CXCR4	(60)	−	−	−	#	#	−
5HT1A–5HT1B	(61)	−	−	−	−	#	#
5HT1A–5HT7	(62)	−	−	−	−	#	#
ADRA2A–ADRA2B	(63)	−	−	−	−	−	#
ADRB2–ADRB3	(64)	−	−	−	−	#	#
D2–CCR2	Possible heteromer	−	−	−	#	−	−
D2–IL1R2	Possible heteromer	−	−	−	−	#	#
D2–CXCR4	Possible heteromer	−	−	−	#	#	−

*#, yes in both receptors and may mediate their interaction; +, yes in both receptors; −, no in any receptor*.

Location: ec, extracellular; ic, intracellular; TM, transmembrane; SVS—ic GABAB1 # CXCR4; ec NR2A,B,D # IL1R2(N-terminal); GLL—TM GABAB1 # GABAB2; ic NR2A # CCR2(C-tail); VST—ic GABAB1 # CXCR4 (C-tail), NR2A # CXCR4(C-tail), NR1-1,4,5(C-tail) # CXCR4(C-tail); LYS—TM MOP, DOP, CXCR4, D2; LPF—TM MOP, DOP, 5HT1A,B, 5HT7; TM CXCR4 # D2(TM’ec); ec IL1R2 # D2(TM’ec); SLA—TM 5HT1A,B, 5HT7, ADRA2A,B, ADRB2,3; TM IL1R2 # D2. The highlighted text in red color represent the new postulated heteroreceptor complexes based on the Triplet Puzzle Theory

Interleukin 1 receptor type II is a decoy receptor that can bind to IL1α, IL1β, and IL1R antagonist. It can also interact with IL1R accessory protein. It should be noticed that the ITGA-ITGB heterodimer shows a SVS protriplet homology and the GABA B receptor (GABAB1-GABAB2 heterodimer) a GLL protriplet homology and the known GABAB1-CXCR4 heterodimer *inter alia* a VST and a SVS homology (Table 1). These observations further support the current view that NMDARs can form heterormers with CCR2, CXCR4, and IL1R2.

**Figure 1:**
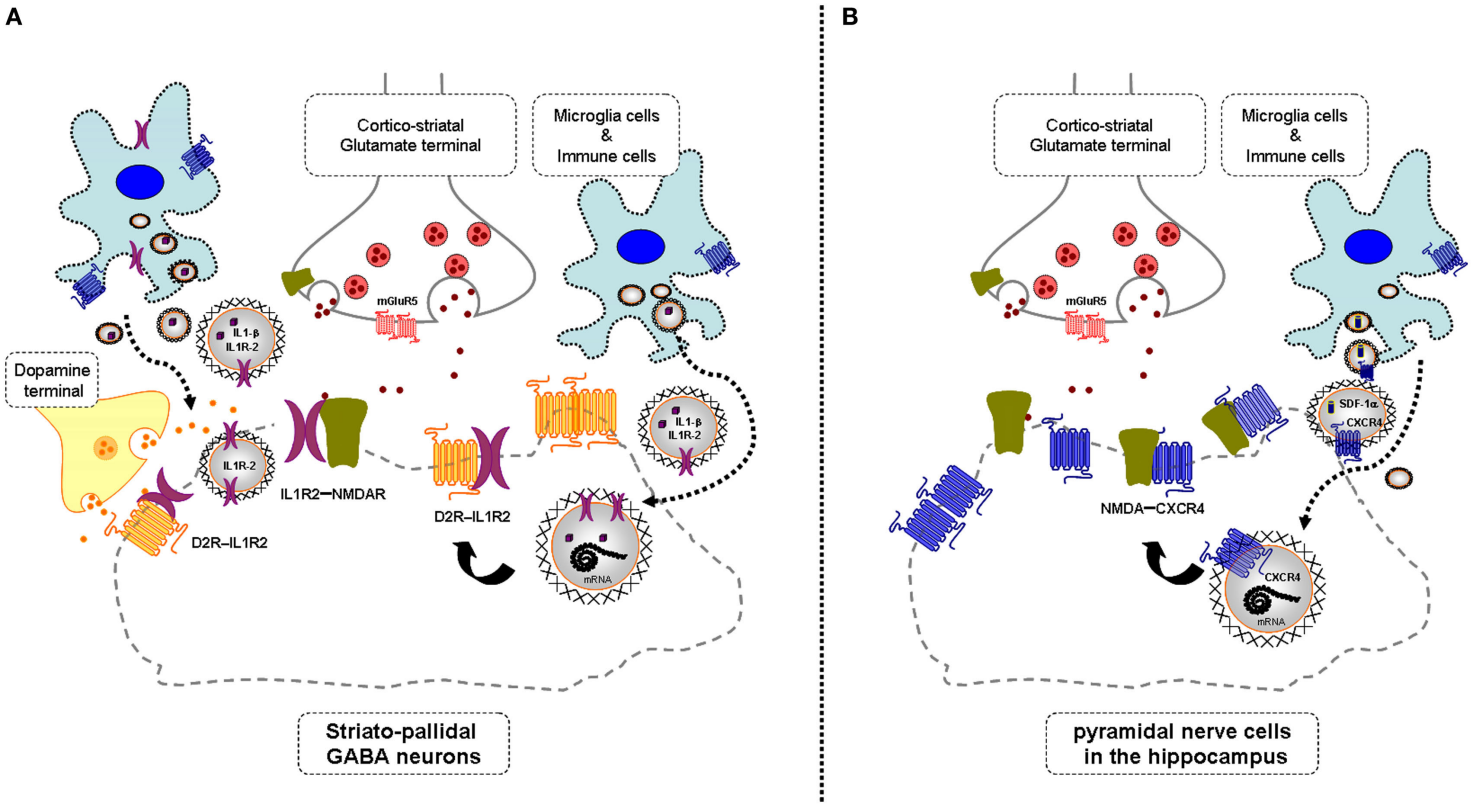
**Illustration of the potential chemokine and cytokine receptor transfer *via* extracellular vesicle-volume transmission (VT) from immune and microglial cells to striatopallidal GABA neurons [containing dopamine (DA) D2Rs and NMDARs] and hippocampus pyramidal neurons (containing NMDA receptors) in mild inflammation**. One mechanism is shown for how chemokine C-X-C chemokine receptor type 4 (CXCR4) and cytokine receptor IL1-R2 including their mRNAs can produce schizophrenia-like symptoms in neuroinflammation. These receptors may be transferred *via* extracellular vesicle-mediated VT from immune cells, activated microglia, and/or astroglia to nerve cells containing DA D2R and NMDA receptors. Upon internalization, the receptors CXCR4 and IL1-R2 can according to the triplet puzzle theory (see Table 1) form complexes with DA D2R and NMDAR as illustrated here. **(A)** is shown the D2R-interleukin 1 receptor type II (IL1R2) heteromers in striatopallidal GABA neurons and how they reach the plasma membrane NMDAR *via* early endosomes and recycling endosomes. **(B)** is shown the NMDAR-CXCR4 heteromers in the pyramidal nerve cells in the hippocampus with microglial extracellular vesicles containing CXCR4 and also SDF-1alpha reaching the NMDAR in the plasma membrane *via* internalization and endosomes in the pyramidal cells. Through the development of novel allosteric receptor–receptor interactions in such heteroreceptor complexes, D2R and NMDAR signaling may become pathologically altered contributing to schizophrenia-like symptoms.

In mild neuroinflammation, it is proposed that VT is increased involving increased extracellular vesicle-mediated VT from microglia and astroglia (see above). It should, therefore, be considered that these vesicles may contain CCR2, CXCR4, and/or IL1R2, which upon internalization into neurons can form heteroreceptor complexes with NMDAR with pathological allosteric receptor–receptor interactions (Figure 1). If these receptor mechanisms lead to a hypofunction of the NMDAR protomer, they represent one mechanism for the schizophrenia-like effects seen in mild neuroinflammation (3). If the mild neuroinflammation takes place in the hippocampus and the cerebral cortex, the pyramidal nerve cells, key nerve cells in the cortical, and hippocampal circuits will also be affected in view of their expression of NMDA receptors. It will lead to deficits in cognitive functions and contribute to negative symptoms of schizophrenia.

These disturbances may become enhanced by an increase in the glia release of the endogenous cytokine and chemokine ligands for these receptors. The agonist induced receptor–receptor interactions can amplify the reduction of NMDAR function in these heterocomplexes and thus help develop the schizophrenia episodes. It will be of high interest to test if the putative NMDAR-CCR2, NMDAR-CXCR4, and NMDAR-IL1R2 heteroreceptor complexes in fact exist using the *in situ* proximity ligation assay in brain tissue. It is postulated that a reduced NMDAR signaling develop in these heteroreceptor complexes through the agonist activated chemokine and cytokine receptor protomers reducing the NMDAR signaling *via* allosteric mechanisms in these heterocomplexes. This may also lead to increases in the internalization of these heterocomplexes to late endosomes and lysosomes with a reduction in the density of NMDA receptors. It is unknown if the cytokine and/or chemokine-activated receptor protomer involves a negative allosteric modulation of the glutamate-binding site on GluN2 and/or a negative allosteric modulation of the glycine modulatory site on GluN1 (25).

C-C chemokine receptor type 2 with its ligand CCL2 plays a major role in immunobiology and neurobiology (65). It is mainly located on monocytes and is involved in systemic and brain inflammation. CXCR4 and its ligands CXCL12 are involved in the pathogenesis of brain disease and participate in neuron–glial interactions and in neurotoxicity (66, 67). The IL1R2 exists both in soluble and membrane bound forms, shows no transmembrane signaling, and is regarded as a decoy receptor for IL1 signaling However, it can also act as a binding protein in the membrane and interact with the IL1R accessory protein (68). This accessory protein also exists as an alternatively spliced brain-specific isoform having a significant role in homeostatic sleep (69).

According to the triplet puzzle hypothesis, it can bind to the NMDAR [Table 1; see also Ref. (51)] and form a NMDAR–IL1R2 complex in which NMDAR function is postulated to become reduced. Previous work (70) indicated that this receptor can participate in Alzheimer’s disease, but its biological function is unknown. It may participate in anti-inflammation and help keep the neuroinflammation at a low level.

### Triplet Puzzle Theory Supports the Formation of Dopamine (DA) D2R–Cytokine Receptor/Chemokine Receptor Heteroreceptor Complexes through LYS, LPF, and SLA Triplet Homologies

There exists dysregulation of the DA neurons in the pathophysiology of schizophrenia (71). The therapeutic effects of typical and atypical antipsychotic drugs are mainly mediated *via* blockade of the DA D2 receptors (72, 73) located postjunctionally in the mesolimbic-cortical DA neurons (74–76). The D2Rs are mainly located outside the DA synapses and targeted by extracellular DA VT (76). Over the last decades, the discovery was made that D2Rs participate in many different types heteroreceptor complexes in which D2R protomers directly interact *via* allosteric mechanisms with other receptor protomers to integrate biological signals changing D2R signaling (47, 49). Some of them offer new targets for the therapeutic effects of D2R antagonists. In other D2R heteroreceptor complexes, the blockade of the D2R protomers by antipsychotic drugs may instead produce side-effects.

It is of particular interest that the NR2B subunit of the NMDAR interacts with the D2R in the glutamate synapses (77), which leads to an antagonistic allosteric receptor–receptor interaction reducing NMDA signaling in the heteroreceptor complex. Thus, DA through VT may diffuse into the glutamate synapses, activate D2R protomers, and reduce NMDAR-mediated synaptic glutamate transmission, which should enhance schizophrenic symptoms according to current hypotheses. The D2Rs and their heteroreceptor complexes are mainly located in the ventral striatopallidal GABA anti-reward neurons. Their enhanced inhibition by enhanced D2R signaling leading to reduced NMDAR signaling should markedly bring down anti-reward activity in this pathway, which can contribute to a malfunction of salience in schizophrenia with all stimuli becoming relevant and disturbing ongoing behavior (49).

It is, therefore, of high interest that, according to the triplet puzzle theory, D2Rs can form heteroreceptor complexes with CCR2, IL1R2, and CXCR4 as indicated for the first time in the current article (Table 1). Three “schizo triplets” were found: LYS, LPF, and SLA. The possible D2R-IL1R2 heterodimer had two sets of triplet homologies, LPF and SLA. LPF is located in the TM6/extracellular region of the D2R and in the extracellular region of the IL1R2 (Table 1). SLA is instead located in TM2 of the D2R and in the TM region of IL1R2 (Table 1). The possible D2R-CXCR4 heterodimer also shows two sets of triplet homologies, one is again LPF, this time located in the interface between TM6-TM2 with the LPF located in the TM2 (Table 1). The other triplet is LYS, which is located in the TM7 of the D2 and in TM3 of the CXCR4 (Table 1) indicating that TM7 and TM3 of the D2R and CXCR4, respectively, can also participate in this interface. The possible D2R-CCR2 heterodimer only exhibits one set of triplet homology that may help mediate the interaction. Also, in this case, the LYS triplet is found and here present in the TM1 of CCR2 that may interact with the LYS triplet in TM7 of D2R.

It is proposed that D2R-CCR2, D2R-IL1R2, and D2R-CXCR4 heteromers can be formed upon mild neuroinflammation in the brain, especially in the ventral striatum. This may contribute to positive schizophrenic symptoms by enhancing D2R inhibitory function in critical brain circuits like the ventral striatopallidal GABA anti-reward pathway leading to exaggerated salience development (Figure 1). Both chemokine and cytokine receptors appear to be involved in forming complexes with the DA D2Rs as is the case with NMDARs. It should also be underlined that the NMDARs interact with the same chemokine and cytokine receptor subtypes as the D2Rs but using different sets of triplet amino acid homologies.

#### Other GPCR Heteroreceptor Complexes with LYS, LPF, and SLA Homologies

It is of interest that a number of 5HT1A isoreceptor complexes and α2- and β-adrenergic isoreceptor complexes (78) also possess sets of LYS, LPF, and SLA protriplets that may assist in the formation of these isoreceptor complexes (Table 1). Previous work demonstrated also crosstalk between opioid and chemokine receptor subtypes (79) and extensive formation of heteromers take place between opioid and receptor subtypes according to the triplet puzzle theory (80). In Table 1, we report some results from this study showing that LYS and LPF protriplets may also participate in opioid and chemokine isoreceptor complexes as well as in the delta opioid-CXCR4 heterodimer. Their possible role in schizophrenic symptoms in neuroinflammation remains to be explored, but they may have an impact on pain and reward mechanisms (80).

### Hypothesis: Glial Cytokine and Chemokine Receptor Subtype Transfer into Neurons in Mild Neuroinflammation Can Produce Novel Dysfunctional NMDAR and D2R Heteroreceptor Complexes Contributing to Schizophrenia Development

This hypothesis is based on the existence of not only soluble VT signals but also of extracellular vesicle-mediated VT signals (44). In 2006, it was found that exosomes can be released from cortical neurons in culture (81). In 2012, cell cultures were demonstrated to transfer GPCRs *via* extracellular vesicles to other cells and also form GPCR heteromers in the recipient cells by direct interactions with their GPCRs and A2AR-D2R heteromers developed (82). Glial cells, especially the microglia, are activated in inflammation and can release a number of chemokines and cytokines as soluble VT signals and produce a panorama of cytokine and chemokine receptors (6, 83). They may communicate as soluble VT signals (ligands) and *via* extracellular vesicle-mediated VT as to receptors and their receptor mRNAs (43, 44). The extracellular vesicles may then *via* cell adhesion receptors become internalized into neurons and their cargo released. In the neurons, CCR2, CXCR4, and IL1R2 can according to the triplet puzzle theory (see above) interact with NMDARs, known to be disturbed in schizophrenia, and as discovered in the current paper with D2Rs, the major target for currently used antipsychotic drugs.

The hypothesis states that the NMDAR protomer develops a hypofunction in mild neuroinflammation due to antagonistic receptor–receptor interactions produced by activation of the CCR2, CXCR4, and/or IL1R2 protomers in the plasma membrane. Their agonist ligands are released by the microglia and/or immune cells and/or astroglia into the extracellular fluid to activate these chemokine and cytokine receptor protomers in the plasma membrane. The current findings based on the triplet puzzle theory indicate that the CCR2, CXCR4, and/or IL1R2 can also interact with D2Rs through such mechanisms. Based on the antipsychotic actions of D2R antagonists, it is proposed that enhancing allosteric receptor–receptor interactions develop in the D2R-CCR2, D2R-CXCR4, and D2R-IL1R2 heteromers upon agonist activation of these chemokine and cytokine receptors leading to increases in D2R protomer signaling with development of schizophrenia-like symptoms.

It will be of high interest to test in cellular models if, in fact, the NMDAR and D2R heteroreceptor complexes with CCR2, CXCR4, and/or IL1R2 protomers are formed using the BRET methodology and the postulated allosteric receptor–receptor interactions develop. We will also test if these NMDAR and D2R heteroreceptor complexes exist in the ventral and dorsal striatum in models of neuroinflammation using the *in situ* proximity ligation assay and if the proposed allosteric receptor–receptor interactions occur in these brain regions upon neuroinflammation with or without agonist ligands for the CCR2, CXCR4, and/or IL1R2 protomers.

## Concluding Remarks

It is proposed that the following mechanisms can contribute to schizophrenia-like symptoms in mild neuroinflammation:

- Extracellular vesicle-mediated VT with receptor and ligand transfer from glial networks to neuronal networks involving distinct cytokine and chemokine receptors and their agonist ligands can lead to formation of dysfunctional and separate NMDAR and D2R heteroreceptor complexes containing CCR2, CXCR4, and/or IL1R2 according to the triplet puzzle theory. The agonist ligands for these three receptors may produce allosteric receptor–receptor interactions in these dysfunctional complexes reducing NMDAR and increasing D2R signaling in the plasma membrane. Schizophrenia-like symptoms may, therefore, develop.
- However, there is no consensus as to which psychopathological symptoms are specific for schizophrenia. There is in fact clear evidence that symptoms in any type of encephalitis are in principle non-specific and variant. As far as the mechanisms proposed in the current paper, they may, from a theoretical perspective, contribute not only to schizophrenia symptoms but may also participate in bipolar disorder and other affective disorders associated with mild neuroinflammation.
- The hypothesis introduced on the formation of distinct NMDAR–cytokine receptor/chemokine receptor and D2R–cytokine receptor/chemokine receptor heterocomplexes with pathological receptor–receptor interactions in the brain upon mild neuroinflammation will primarily be tested as follows: the possible existence of the putative and distinct NMDAR and D2R heteroreceptor complexes will be studied in cellular models and brain models of neuroinflammation using proximity ligation assay and BRET. Then, it will be tested in these models if their allosteric receptor–receptor interactions will lead to a reduction of NMDAR signaling and to increases in D2R signaling in the above heteroreceptor complexes. Finally, if positive results are obtained, the critical role of the demonstrated sets of triplet amino acid homologies for the formation of these heteroreceptor complexes and their receptor–receptor interactions will be tested through mutations of these triplet homologies.

## Author Contributions

KF made substantial contributions to the conception and design of the work. He participated in the manuscript writing and critically evaluated it. He agreed with the submission to Frontiers of the current version. DOB-E made substantial contributions to the conception and design of the work, specially the design and conception of the figures. He prepared the reference list and participated in the manuscript writing and critically evaluated it. He agreed with the submission to Frontiers of the current version. AOT made substantial contributions to the conception and design of the work, specially the mathematical and bioinformatic analysis of the amino acid protriplets. He participated in the manuscript writing and critically evaluated it. He agreed with the submission to Frontiers of the current version. KB made substantial contributions to the conception and design of the work. He participated in the manuscript writing and critically evaluated it. He agreed with the submission to Frontiers of the current version.

## Conflict of Interest Statement

The authors have no affiliations or financial involvement with any organization or entity with a financial interest in or financial conflict with the subject matter or materials discussed in the manuscript. The reviewer MZ and handling Editor declared their shared affiliation, and the handling Editor states that the process nevertheless met the standards of a fair and objective review.

## References

[1] Bechter K Mild encephalitis underlying psychiatric disorder – a reconsideration and hypothesis examplified on Borna disease. Neurol Psychiatry Brain Res (2001) 9:55–70.

[2] Bechter K [The mild encephalitis-hypothesis – new findings and studies]. Psychiatr Prax (2004) 31(Suppl 1):S41–3. 10.1055/s-2004-828428 15570497

[3] Bechter K [Schizophrenia – a mild encephalitis?]. Fortschr Neurol Psychiatr (2013) 81:250–9. 10.1055/s-0033-1335253 23629631

[4] Muller N Weidinger E Leitner B Schwarz MJ The role of inflammation in schizophrenia. Front Neurosci (2015) 9:372 10.3389/fnins.2015.00372 26539073 PMC4612505

[5] Hong H Kim BS Im HI. Pathophysiological role of neuroinflammation in neurodegenerative diseases and psychiatric disorders. Int Neurourol J (2016) 20:S2–7. 10.5213/inj.1632604.302 27230456 PMC4895907

[6] Kettenmann H Hanisch UK Noda M Verkhratsky A. Physiology of microglia. Physiol Rev (2011) 91:461–553. 10.1152/physrev.00011.2010 21527731

[7] Kettenmann H Kirchhoff F Verkhratsky A. Microglia: new roles for the synaptic stripper. Neuron (2013) 77:10–8. 10.1016/j.neuron.2012.12.023 23312512

[8] Inta D Lang UE Borgwardt S Meyer-Lindenberg A Gass P. Microglia activation and schizophrenia: lessons from the effects of minocycline on postnatal neurogenesis, neuronal survival and synaptic pruning. Schizophr Bull (2016). 10.1093/schbul/sbw088 27352782 PMC5464012

[9] Maxeiner HG Rojewski MT Schmitt A Tumani H Bechter K Schmitt M Flow cytometric analysis of T cell subsets in paired samples of cerebrospinal fluid and peripheral blood from patients with neurological and psychiatric disorders. Brain Behav Immun (2009) 23:134–42. 10.1016/j.bbi.2008.08.003 18771722

[10] Maxeiner HG Rojewski MT Tumani H Herzog S Fuchs D Schmitt A Immunological and histochemical analyses of cerebrospinal fluid and peripheral blood from patients with neurological and psychiatric disorders. Acta Neuropsychiatr (2009) 21(Suppl 2):51–7. 10.1017/S0924270800032737 25384871

[11] Bechter K Reiber H Herzog S Fuchs D Tumani H Maxeiner HG. Cerebrospinal fluid analysis in affective and schizophrenic spectrum disorders: identification of subgroups with immune responses and blood-CSF barrier dysfunction. J Psychiatr Res (2010) 44:321–30. 10.1016/j.jpsychires.2009.08.008 19796773

[12] Kuehne LK Reiber H Bechter K Hagberg L Fuchs D Cerebrospinal fluid neopterin is brain-derived and not associated with blood-CSF barrier dysfunction in non-inflammatory affective and schizophrenic spectrum disorders. J Psychiatr Res (2013) 47:1417–22. 10.1016/j.jpsychires.2013.05.027 23790260

[13] Maxeiner HG Marion Schneider E Kurfiss ST Brettschneider J Tumani H Bechter K. Cerebrospinal fluid and serum cytokine profiling to detect immune control of infectious and inflammatory neurological and psychiatric diseases. Cytokine (2014) 69:62–7. 10.1016/j.cyto.2014.05.008 25022963

[14] Bechter K CSF diagnostics in psychiatry – present status – future projects. Neurol Psychiatry Brain Res (2016) 22:69–74. 10.1016/j.npbr.2016.01.008

[15] Bechter K Frequent minor CSF pathologies strongly support the mild encephalitis hypothesis – specific analysis may suggest individual treatment approaches. Neurol Psychiatry Brain Res (2016) 22:1 10.1016/j.npbr.2015.12.003

[16] Bechter K Updating the mild encephalitis hypothesis of schizophrenia. Prog Neuropsychopharmacol Biol Psychiatry (2013) 42:71–91. 10.1016/j.pnpbp.2012.06.019 22765923

[17] Dalmau J Gleichman AJ Hughes EG Rossi JE Peng X Lai M Anti-NMDA-receptor encephalitis: case series and analysis of the effects of antibodies. Lancet Neurol (2008) 7:1091–8. 10.1016/S1474-4422(08)70224-2 18851928 PMC2607118

[18] Dalmau J Lancaster E Martinez-Hernandez E Rosenfeld MR Balice-Gordon R. Clinical experience and laboratory investigations in patients with anti-NMDAR encephalitis. Lancet Neurol (2011) 10:63–74. 10.1016/S1474-4422(10)70253-2 21163445 PMC3158385

[19] Benros ME Mortensen PB Eaton WW. Autoimmune diseases and infections as risk factors for schizophrenia. Ann N Y Acad Sci (2012) 1262:56–66. 10.1111/j.1749-6632.2012.06638.x 22823436

[20] Benros ME Eaton WW Mortensen PB. The epidemiologic evidence linking autoimmune diseases and psychosis. Biol Psychiatry (2014) 75:300–6. 10.1016/j.biopsych.2013.09.023 24199668 PMC8797267

[21] Benros ME Trabjerg BB Meier S Mattheisen M Mortensen PB Mors O Influence of polygenic risk scores on the association between infections and schizophrenia. Biol Psychiatry (2016) 80:609–16. 10.1016/j.biopsych.2016.04.008 27364036

[22] Endres D Perlov E Baumgartner A Hottenrott T Dersch R Stich O Immunological findings in psychotic syndromes: a tertiary care hospital’s CSF sample of 180 patients. Front Hum Neurosci (2015) 9:476. 10.3389/fnhum.2015.00476 26441585 PMC4564575

[23] Endres D Dersch R Hottenrott T Perlov E Maier S Van Calker D Alterations in cerebrospinal fluid in patients with bipolar syndromes. Front Psychiatry (2016) 7:194 10.3389/fpsyt.2016.00194 28008318 PMC5144108

[24] Endres D Perlov E Dersch R Baumgartner A Hottenrott T Berger B Evidence of cerebrospinal fluid abnormalities in patients with depressive syndromes. J Affect Disord (2016) 198:178–84. 10.1016/j.jad.2016.03.030 27017374

[25] Balu DT. The NMDA receptor and schizophrenia: from pathophysiology to treatment. Adv Pharmacol (2016) 76:351–82. 10.1016/bs.apha.2016.01.006 27288082 PMC5518924

[26] Rosi S Ramirez-Amaya V Hauss-Wegrzyniak B Wenk GL. Chronic brain inflammation leads to a decline in hippocampal NMDA-R1 receptors. J Neuroinflammation (2004) 1:12. 10.1186/1742-2094-1-12 15285803 PMC500869

[27] Ma J Choi BR Chung C Min SS Jeon WK Han JS. Chronic brain inflammation causes a reduction in GluN2A and GluN2B subunits of NMDA receptors and an increase in the phosphorylation of mitogen-activated protein kinases in the hippocampus. Mol Brain (2014) 7:33. 10.1186/1756-6606-7-33 24761931 PMC4021635

[28] Vincent A Bien CG Anti-NMDA-receptor encephalitis: a cause of psychiatric, seizure, and movement disorders in young adults. Lancet Neurol (2008) 7:1074–5. 10.1016/S1474-4422(08)70225-4 18851929

[29] Zandi MS Irani SR Lang B Waters P Jones PB McKenna P Disease-relevant autoantibodies in first episode schizophrenia. J Neurol (2011) 258:686–8. 10.1007/s00415-010-5788-9 20972895 PMC3065649

[30] Pruss H Finke C Holtje M Hofmann J Klingbeil C Probst C N-methyl-d-aspartate receptor antibodies in herpes simplex encephalitis. Ann Neurol (2012) 72:902–11. 10.1002/ana.23689 23280840 PMC3725636

[31] Hughes EG Peng X Gleichman AJ Lai M Zhou L Tsou R Cellular and synaptic mechanisms of anti-NMDA receptor encephalitis. J Neurosci (2010) 30:5866–75. 10.1523/JNEUROSCI.0167-10.2010 20427647 PMC2868315

[32] Kreye J Wenke NK Chayka M Leubner J Murugan R Maier N Human cerebrospinal fluid monoclonal N-methyl-d-aspartate receptor autoantibodies are sufficient for encephalitis pathogenesis. Brain (2016) 139:2641–52. 10.1093/brain/aww208 27543972

[33] Pruss H. [Pathophysiology and prognostic factors of autoimmune encephalitis]. Fortschr Neurol Psychiatr (2016) 84:264–70. 10.1055/s-0041-109127 27299785

[34] Najjar S Pearlman D Devinsky O Najjar A Nadkarni S Butler T Neuropsychiatric autoimmune encephalitis without VGKC-complex, NMDAR, and GAD autoantibodies: case report and literature review. Cogn Behav Neurol (2013) 26:36–49. 10.1097/WNN.0b013e31828b6531 23538571

[35] Najjar S Pearlman DM Alper K Najjar A Devinsky O Neuroinflammation and psychiatric illness. J Neuroinflammation (2013) 10:43 10.1186/1742-2094-10-43 23547920 PMC3626880

[36] Najjar S Diagnostic and therapeutic dilemmas inherent to neuropsychiatric disorders associated with neuroinflammation or autoimmune dysfunction. In: PAP German Association for Psychiatry, editor. DGPPN Kongress. Berlin, Germany: Globit GmbH (2016). p. 96.

[37] Bogerts B Winopal D Schwarz S Schlaaff K Dobrowolny H Mawrin C Evidence of neuroinflammation in subgroups of schizophrenia and mood disorder patients: a semiquantitative postmortem study of CD3 and CD20 immunoreactive lymphocytes in several brain regions. Neurol Psychiatry Brain Res (2017) 23:2–9. 10.1016/j.npbr.2016.11.001

[38] Coutinho E Harrison P Vincent A. Do neuronal autoantibodies cause psychosis? A neuroimmunological perspective. Biol Psychiatry (2014) 75:269–75. 10.1016/j.biopsych.2013.07.040 24090795

[39] Brown DA Sawchenko PE. Time course and distribution of inflammatory and neurodegenerative events suggest structural bases for the pathogenesis of experimental autoimmune encephalomyelitis. J Comp Neurol (2007) 502:236–60. 10.1002/cne.21307 17348011

[40] Lucchinetti CF Popescu BF Bunyan RF Moll NM Roemer SF Lassmann H Inflammatory cortical demyelination in early multiple sclerosis. N Engl J Med (2011) 365:2188–97. 10.1056/NEJMoa1100648 22150037 PMC3282172

[41] Parpura V Heneka MT Montana V Oliet SH Schousboe A Haydon PG Glial cells in (patho)physiology. J Neurochem (2012) 121:4–27. 10.1111/j.1471-4159.2012.07664.x 22251135 PMC3304021

[42] Aguzzi A Barres BA Bennett ML. Microglia: scapegoat, saboteur, or something else? Science (2013) 339:156–61. 10.1126/science.1227901 23307732 PMC4431634

[43] Fuxe K Borroto-Escuela DO Tarakanov AO Romero-Fernandez W Manger P Rivera A Understanding the balance and integration of volume and synaptic transmission. Relevance for psychiatry. Neurol Psychiatr Brain Res (2013) 19:141–58. 10.1016/j.npbr.2013.10.002

[44] Borroto-Escuela DO Agnati LF Bechter K Jansson A Tarakanov AO Fuxe K. The role of transmitter diffusion and flow versus extracellular vesicles in volume transmission in the brain neural-glial networks. Philos Trans R Soc Lond B Biol Sci (2015) 370:20140183. 10.1098/rstb.2014.0183 26009762 PMC4455752

[45] Mulcahy LA Pink RC Carter DR. Routes and mechanisms of extracellular vesicle uptake. J Extracell Vesicles (2014) 3:1–14. 10.3402/jev.v3.24641 25143819 PMC4122821

[46] Jean-Alphonse F Hanyaloglu AC. Regulation of GPCR signal networks via membrane trafficking. Mol Cell Endocrinol (2011) 331:205–14. 10.1016/j.mce.2010.07.010 20654691

[47] Fuxe K Borroto-Escuela DO Tarakanov AO Romero-Fernandez W Ferraro L Tanganelli S Dopamine D2 heteroreceptor complexes and their receptor-receptor interactions in ventral striatum: novel targets for antipsychotic drugs. Prog Brain Res (2014) 211:113–39. 10.1016/B978-0-444-63425-2.00005-2 24968778

[48] Fuxe K Tarakanov A Romero Fernandez W Ferraro L Tanganelli S Filip M Diversity and bias through receptor-receptor interactions in GPCR heteroreceptor complexes. Focus on examples from dopamine D2 receptor heteromerization. Front Endocrinol (2014) 5:71 10.3389/fendo.2014.00071 PMC4026686 24860548

[49] Borroto-Escuela DO Pintsuk J Schafer T Friedland K Ferraro L Tanganelli S Multiple D2 heteroreceptor complexes: new targets for treatment of schizophrenia. Ther Adv Psychopharmacol (2016) 6:77–94. 10.1177/2045125316637570 27141290 PMC4837969

[50] Tarakanov AO Fuxe KG. Triplet puzzle: homologies of receptor heteromers. J Mol Neurosci (2010) 41:294–303. 10.1007/s12031-009-9313-5 19960372

[51] Tarakanov AO Fuxe KG. Integrin triplets of marine sponges in the murine and human MHCI-CD8 interface and in the interface of human neural receptor heteromers and subunits. Springerplus (2013) 2:128. 10.1186/2193-1801-2-128 23556147 PMC3612178

[52] Tarakanov AO Fuxe KG Borroto-Escuela DO Integrin triplets of marine sponges in human brain receptor heteromers. J Mol Neurosci (2012) 48:154–60. 10.1007/s12031-012-9793-6 22573093

[53] Tarakanov AO Fuxe KG Borroto-Escuela DO Integrin triplets of marine sponges in human D2 receptor heteromers. J Recept Signal Transduct Res (2012) 32:202–8. 10.3109/10799893.2012.692119 22712841

[54] White JH Wise A Main MJ Green A Fraser NJ Disney GH Heterodimerization is required for the formation of a functional GABA(B) receptor. Nature (1998) 396:679–82. 10.1038/25354 9872316

[55] Billinton A Ige AO Wise A White JH Disney GH Marshall FH GABA(B) receptor heterodimer-component localisation in human brain. Brain Res Mol Brain Res (2000) 77:111–24. 10.1016/S0169-328X(00)00047-4 10814837

[56] Marshall FH Heterodimerization of G-protein-coupled receptors in the CNS. Curr Opin Pharmacol (2001) 1:40–4. 10.1016/S1471-4892(01)00001-7 11712533

[57] Guyon A Nahon JL. Multiple actions of the chemokine stromal cell-derived factor-1alpha on neuronal activity. J Mol Endocrinol (2007) 38:365–76. 10.1677/JME-06-0013 17339399

[58] Gomes I Jordan BA Gupta A Trapaidze N Nagy V Devi LA. Heterodimerization of mu and delta opioid receptors: a role in opiate synergy. J Neurosci (2000) 20:RC110. 11069979 10.1523/JNEUROSCI.20-22-j0007.2000 PMC3125672

[59] Percherancier Y Berchiche YA Slight I Volkmer-Engert R Tamamura H Fujii N Bioluminescence resonance energy transfer reveals ligand-induced conformational changes in CXCR4 homo- and heterodimers. J Biol Chem (2005) 280:9895–903. 10.1074/jbc.M411151200 15632118

[60] Pello OM Martinez-Munoz L Parrillas V Serrano A Rodriguez-Frade JM Toro MJ Ligand stabilization of CXCR4/delta-opioid receptor heterodimers reveals a mechanism for immune response regulation. Eur J Immunol (2008) 38:537–49. 10.1002/eji.200737630 18200497

[61] Salim K Fenton T Bacha J Urien-Rodriguez H Bonnert T Skynner HA Oligomerization of G-protein-coupled receptors shown by selective co-immunoprecipitation. J Biol Chem (2002) 277:15482–5. 10.1074/jbc.M201539200 11854302

[62] Renner U Zeug A Woehler A Niebert M Dityatev A Dityateva G Heterodimerization of serotonin receptors 5-HT1A and 5-HT7 differentially regulates receptor signalling and trafficking. J Cell Sci (2012) 125:2486–99. 10.1242/jcs.101337 22357950

[63] Zhou F Filipeanu CM Duvernay MT Wu G Cell-surface targeting of alpha2-adrenergic receptors – inhibition by a transport deficient mutant through dimerization. Cell Signal (2006) 18:318–27. 10.1016/j.cellsig.2005.05.014 15961277 PMC2718052

[64] Breit A Lagace M Bouvier M. Hetero-oligomerization between beta2- and beta3-adrenergic receptors generates a beta-adrenergic signaling unit with distinct functional properties. J Biol Chem (2004) 279:28756–65. 10.1074/jbc.M313310200 15123695

[65] Yamasaki R Lu H Butovsky O Ohno N Rietsch AM Cialic R Differential roles of microglia and monocytes in the inflamed central nervous system. J Exp Med (2014) 211:1533–49. 10.1084/jem.20132477 25002752 PMC4113947

[66] Ramesh G Maclean AG Philipp MT Cytokines and chemokines at the crossroads of neuroinflammation, neurodegeneration, and neuropathic pain. Mediators Inflamm (2013) 2013:480739 10.1155/2013/480739 23997430 PMC3753746

[67] Ramesh G Philipp MT Vallieres L Maclean AG Ahmad M Mediators of neuroinflammation. Mediators Inflamm (2013) 2013:314261 10.1155/2013/314261 24222936 PMC3816058

[68] Hanawa H Ota Y Ding L Chang H Yoshida K Otaki K IL-1 receptor accessory protein-Ig/IL-1 receptor type II-Ig heterodimer inhibits IL-1 response more strongly than other IL-1 blocking biopharmaceutical agents. J Clin Immunol (2011) 31:455–64. 10.1007/s10875-010-9497-z 21181432

[69] Davis CJ Dunbrasky D Oonk M Taishi P Opp MR Krueger JM. The neuron-specific interleukin-1 receptor accessory protein is required for homeostatic sleep and sleep responses to influenza viral challenge in mice. Brain Behav Immun (2015) 47:35–43. 10.1016/j.bbi.2014.10.013 25449578 PMC4418942

[70] Garlind A Brauner A Hojeberg B Basun H Schultzberg M. Soluble interleukin-1 receptor type II levels are elevated in cerebrospinal fluid in Alzheimer’s disease patients. Brain Res (1999) 826:112–6. 10.1016/S0006-8993(99)01092-6 10216202

[71] Grace AA. Dysregulation of the dopamine system in the pathophysiology of schizophrenia and depression. Nat Rev Neurosci (2016) 17:524–32. 10.1038/nrn.2016.57 27256556 PMC5166560

[72] Seeman P Historical overview. Introduction to the dopamine receptors. In: Neve K, editor. The Dopamine Receptors. New York: Humana Press (2010). p. 1–21.

[73] Ginovart N Kapur S. Role of dopamine D(2) receptors for antipsychotic activity. Handb Exp Pharmacol (2012) 212:27–52. 10.1007/978-3-642-25761-2_2 23129327

[74] Fuxe K Anden NE Studies on the central monoamine neurons with special reference to the nigro-neostriatal dopamine neuron system. In: Costa E Coté L Yahr M, editors. Biochemistry and Pharmacology of the Basal Ganglia. New York: Raven Press (1965). p. 123–9.

[75] Thierry AM Blanc G Sobel A Stinus L Glowinski J. Dopaminergic terminals in the rat cortex. Science (1973) 182:499–501. 10.1126/science.182.4111.499 4744179

[76] Fuxe K Dahlstrom A Hoistad M Marcellino D Jansson A Rivera A From the Golgi-Cajal mapping to the transmitter-based characterization of the neuronal networks leading to two modes of brain communication: wiring and volume transmission. Brain Res Rev (2007) 55:17–54. 10.1016/j.brainresrev.2007.02.009 17433836

[77] Liu XY Chu XP Mao LM Wang M Lan HX Li MH Modulation of D2R-NR2B interactions in response to cocaine. Neuron (2006) 52:897–909. 10.1016/j.neuron.2006.10.011 17145509

[78] Borroto-Escuela DO Brito I Romero-Fernandez W Di Palma M Oflijan J Skieterska K The G protein-coupled receptor heterodimer network (GPCR-HetNet) and its hub components. Int J Mol Sci (2014) 15:8570–90. 10.3390/ijms15058570 24830558 PMC4057749

[79] Rostene W Kitabgi P Parsadaniantz SM. Chemokines: a new class of neuromodulator? Nat Rev Neurosci (2007) 8:895–903. 10.1038/nrn2255 17948033

[80] Tarakanov AO Fuxe K. The triplet puzzle theory indicates extensive formation of heteromers between opioid and chemokine receptor subtypes. J Neural Transm (Vienna) (2015) 122:1509–14. 10.1007/s00702-015-1421-5 26133164

[81] Faure J Lachenal G Court M Hirrlinger J Chatellard-Causse C Blot B Exosomes are released by cultured cortical neurones. Mol Cell Neurosci (2006) 31:642–8. 10.1016/j.mcn.2005.12.003 16446100

[82] Guescini M Leo G Genedani S Carone C Pederzoli F Ciruela F Microvesicle and tunneling nanotube mediated intercellular transfer of g-protein coupled receptors in cell cultures. Exp Cell Res (2012) 318:603–13. 10.1016/j.yexcr.2012.01.005 22266577

[83] Pocock JM Kettenmann H. Neurotransmitter receptors on microglia. Trends Neurosci (2007) 30:527–35. 10.1016/j.tins.2007.07.007 17904651

